# The Biochemistry of Pellagra

**Published:** 1919-02

**Authors:** E. M. Perdue

**Affiliations:** Professor of Tropical Medicine, Eclectic Medical University, Kansas City, Mo.


### ORIGINAL ARTICLES.

### The Biochemistry of Pellagra.

BY E. M. PERDUE, A. M., M. D., D. P. H.,
Professor of Tropical Medicine, Eclectic Medical University,
Kansas City, Mo.

A careful review of the literature of the past few years is very suggestive of the fact that the biochemists as well as many others have been led into serious error in classifying pellagra with the "deficiency diseases." This is such an important matter in view of the prevalence and mortality of pellagra, and its ready prevention and cure, that it should not pass without attention.

There are several reasons why the biochemists and others have been deceived and led into this error.

1. The various theories based upon the general maize diet theory had enjoyed wide acceptance, largely through the commanding genius of Lombroso.

2. From 1910 to 1914, in three preliminary notes and several monographs, Alessandrini and Scala of the Institute of Experimental Hygiene of the University of Rome, under the direction of Celli and the patronage of Cencelli, had seriously attacked the maize and diet theories and had finally overthrown them entirely, and in 1913 and 1914, established the true cause of pellagra.

3. Certain large, powerful and influential institutions and agencies, under what Reed has so aptly termed a "policy of concerted and cumulative negation" had initiated a series of "counter researches" whose end and purpose was to discredit the proof and findings of Alessandrini and Scala.

4. The negative findings of these researches, previously agreed upon, were published in certain well known and "authoritative" medical and scientific journals and in the Reports of the United States Public Health Service. His last especially enjoys the prestige and frank of the government.

5. The biochemists, who were not doing special reseach work in pellagra, took these reports in good faith and interpreted them at their face value.

6. Many others, for the same reasons, have been deceived and have written of pellagra as a disease caused by a faulty and unbalanced diet.

Hence the inclusion of pellagra in the list of "deficiency diseases," grossly erroneous though it may be. It is only fair to state that this does not appear to be the fault of the biochemists, nor of others who have been deceived and misled by those immediately concerned in research in pellagra.

A study of the references and bibliographies appended to a number of important articles by leading biochemists not only confirms number 5 of the foregoing statements, but establishes the further fact that the rather extensive literature leading to the demonstration of the true cause of pellagra has been overlooked or ignored. A charitable view of the matter would suggest that it had been overlooked. It has been ignored purposely by the "counter research" in pellagra. A striking example of how a sincere biochemist has been misled may be found in the excellent resume by Casimir Funk in the June-September, 1915, Biochemical Bulletin, pages 326 to 333. It will be noted that nowhere in his article on the history of pellagra research, nor in his bibliography, does he mention the research of Alessandrini and Scala, the clearing up of pellagra in Italy by the improvement of the water supply, or the universal success attained all over the world in curing pellagra by the methods suggested by the Italian scientists.

From time to time, articles have appeared in the medical press referring to the researches of the biochemists as demonstrating the theories of insufficient and unbalanced diet as etiological factors in pellagra. Recently there has been a recrudescence of this discussion in Journal of the A. M. A., September 21, 1918, pages 937 to 956. It will be noted that these lengthy articles made no reference to the research of Alessandrini and Scala and that there is no quotation of these authorities in the references appended as footnotes. This all tends to mislead the profession as to the true cause and prevention of pellagra. The profession is entitled to know these things and the suffering public in the pellagrous zone is entitled to the benefit of such knowledge. The altruism and humanity of the profession of medicine demands nothing less. Hence this article.

### REVIEW OF RESEARCH.

In 1903, the Bureau of Soils of the Department of Agriculture found that the soils of Spartanburg County, South Carolina, were residual clays derived from the weathering and disintegration of the rocks of the Piedmont Plateau. "Observations made in wells and deep cuts show that the soil depth upon the parent rock varies from 25 to 50 feet. The rocks themselves, consisting for the most part of granites and gneisses, become coarser grained as they reach the mountains, often having the appearance of conglomerate. They are among the oldest of rocks."—Soil Survey of the Campobella Area, South Carolina. Field Operations of the Bureau of Soils, 1903, page 303.

In 1909, Buchanan, Superintendent of the State Hospital, Meridian, Mississippi, in speaking of the pellagrous patients in that institution, said:

"All were country people—fairly well-to-do, living in open houses, and using good spring or well water. While they did not come from any particular section, all came from the hilly and sandy regions and none were from the delta or prairies."—Transactions of National Conference on Pellagra, Columbia, South Carolina, November 3, 4, 1909.

Similar observations were made by Dr. J. Roddey Miller, of Rock Hill, South Carolina. Many others locate the prevalence of the disease on the Piedmont Plateau or the Coastal Plain.

In 1910, Alessandrini stated:

"le frazioni piu infestate sono quelle del piano e delle colline costituite da terreni per la massima parte argillosi."

"Queste ragioni mi confermarano nella mia idea e mi convinsero cercar nelle acqua l'agente della pellagra."—Policlinico, Sez. Pratica, Roma, 15 maggio, 1910.

"the divisions most infested are those of the plain and the hills composed of a soil for the greater part clay."

"These reasons confirmed me in my idea and determined me to search in the water the agent of pellagra."

In a later communication Alessandrini stated:

"senza tema di errare, si puo affermare che la malattia esiste solo o nelle zone formate da colli o nel piano ed *e' scarsissima o manca del tutto nelle regioni montuose.*"—Policlinico, Sez. Pratica, 21 giugno, 1910.

"without fear of error, it is possible to state that the disease exists only either in the zone formed by hills or in the plain and is very rare or totally wanting in mountainous regions."

In his monograph, "Sulla Pellagra in Italia," in Annali d'Igiene Sperimentale, 1910, at page 408, Alessandrini says:

"i dati epidemiologi le numerose osservazioni, le prove che ho potuto raccogliere, la concordanza di esse mi han persuaso che *la pellagra e' una malattia causata dall' ingestione di determinate acqua.*"

"the epidemiological data, the numerous observations, the proof which I have been able to collect, the agreement of these have persuaded me that *pellagra is a disease caused by the ingestion of determinate water.*"

In the same monograph at page 420 he comes to this conclusion for the following distinct and scientific reasons. We translate from the original Italian.

1. The absolute absence of the disease where limpid running water is drunk;

2. Its presence where stagnant water is used derived from ponds and wells in superficial strata:

3. The gradual and slow disappearance of the disease where there has been a gradual and slow improvement in the water supply;

4. Its rapid disappearance where aqueducts have been constructed or at the same time numerous artesian wells have been bored, wells in deep strata, or wells already existing and in bad condition have been placed in good repair;

5. The obstinate persistence or the progressive increase of the disease where nothing has been done with respect to the drinking water;

6 The presence of pellagra in localities where the water is good is limited to individuals who emigrated from localities where the water is bad where they contracted the disease;

7. The greater and almost exclusive presence of pellagra in the country, especially where the population is more dense and where little or nothing has been done to improve the condition of the drinking water.

At the second triennial meeting of the National Association for Study of Pellagra, held at Columbia, South Carolina, October 3-4, 1912, Dr. C. W. Rohrer of the Maryland State Department of Health, read a paper on Pellagra in Maryland, which was published in the Virginia Medical Semi-Monthly, November 22, 1912. In this paper Dr. Rohrer makes the following illuminating statement:

"Down to the present writing (September 25, 1912), there have been 31 cases of pellagra accredited to the State of Maryland. Nine of these individuals developed the disease in the City of Baltimore, while 22 were county cases. All have occurred in the Coastal Plain and in the Piedmont Plateau, where the rainfall is greatest and the soil composed of sands, clays, loams and marls. Not a single instance of the disease has been found in the higher, drier, more elevated portion of the State, known as Appalachian Region."

The Pellagrological Commission of the Province of Rome, at the sitting of the 4th of June, 1912, decided to appoint a sub-commission to study pellagra in the province. This sub-commission consisted of Alessandrini, Giannelli and Fileni. They made an extensive investigation and published their report in Policlinico in 1913. They found that the individual cases of pellagra in the province of Rome were invariably associated with drinking of bad water.

Under date of May 22, 1913, Alessandrini and Scala, of the Institute of Experimental Hygiene of the University of Rome, published their Preliminary Note in which they announced the true cause of pellagra. This preliminary note was entitled "Contributo nuovo alla etiologia e patogenesi della pellagra," "Nota preventiva." It was published in Bolletino della R. Accademia Medica di Roma, Anno XXXIX, Fasciolo VII, 1913. This preliminary note was sent to Perdue of Kansas City, Missouri, who translated it into English and published it in Bulletin 1, of the Eclectic Medical University. In this preliminary note Alessandrini and Scala stated:

1. "Pellagra is a disease strictly localized and contracted in a determinate zone where water is constantly drunk which originates in an argillaceous terrain, or flows through or stands on an argillaceous terrain."

2. "Pellagra is the effect of a chronic intoxication which is caused by silica in colloidal solution in water of determinate composition."

They further stated that pellagra is prevented by drinking water containing the proper amount of calcium carbonate, and that pellagra is cured by the administration of an alkaline carbonate. For this purpose they used daily hypodermic injections of one cubic centimeter of a ten per cent solution of neutral sodium citrate. Under this treatment pellagra patients recovered without change of diet, labor, domicile or sanitary environment. They recommend the treatment of public water supplies with broken limestone.

In 1914, Alessandrini and Scala finished their research and published their monograph with the same title as the preliminary note. They sent this monograph at once to Dr. Perdue, at Kansas City, Missouri, with the request that he translate and publish it in English. For this purpose they furnished the original plates of illustration. In this monograph they detail the research by which they arrived at the conclusions stated in the preliminary note. They restate these conclusions and emphasize them. The research detailed in this monograph leaves nothing to be desired in the matter of experimental and clinical proof of the etiology, prevention and cure of pellagra.

For four years Perdue has collaborated with Alessandrini and Scala. He has done the work in Kansas City, Missouri. He has published the results of his work, always giving credit to the Italian scientists, in American Journal of Clinical Medicine, New Orleans Medical and Surgical Journal, Ellingwood's Therapeutist, Homeopathic Recorder, Western Medical Times, Journal of the American Association of Progressive Medicine, Texas Medical Journal, Eclectic Medical Journal, Charlotte Medical Journal and Annali d'Igiene.

In the Western Medical Times, November, 1915, Tichenor of New Orleans, quotes Alessandrini and Scala, Harris and Perdue with approval, states that the cause and cure of pellagra have been discovered, and calls attention to the fact that all real cures of pellagra provide the proper alkalinity.

In 1896, Alessandrini and Scala, in Policlinico, in an article entitled "Risultati di alcune esperienze curative della pellagra con citrato trisodico," published a report of the successful treatment of pellagra in Italy by the hypodermic injection of one cubic centimeter of a ten per cent solution of trisodium citrate. The injections were given daily at first, later on alternate days. In the same journal Dr. Arturo Secchieri reports a series of cases successfully treated with trisodium citrate. The title of his article is "La cura della pellagra con le iniezioni di citrato trisodico."

In Texas Medical Journal, September, 1916, Dr. W. L. Lee, of Hattiesburg, Mississippi, states that colloidal silica is the cause of pellagra, that it is no longer a theory but a demonstrated fact; that all cases can be cured by the alkaline treatment unless the nervous system is so far gone as to be beyond recovery.

In the Lancet, London, April 17, 1915, Cencelli reviews the work of Alessandrini and Scala with approval and outlines the preventive measures taken in Italy.

In the papers already cited, Perdue has shown that pellagra in the United States is strictly localized and contracted only in those regions where the soils are clays derived from the weathering and distintegration of the igneous, crystalline and metamorphic rocks. That these regions are known as the Atlantic and Gulf Coastal Plains, the Piedmont and Ashland Plateaus, the highland of the Ozark, Boston, Oachita, Arbuckle and Wichita mountains in Missouri, Arkansas and Oklahoma; the regions whose soils are derived from the carboniferous shales in Kentucky and Tennessee; the regions covered by glacial drift; the Pacific Coastal Plain. The distribution of pellagra coincides with the distribution of pine timber. Prairies whose treelessness is due to lime in the soils are immune. Where the soils are high in sulfate of lime, or where water supplies are hard because of sulfate or chlorid of lime there may be pellagra. In Annali d'Igiene, Anno XXVI, fasc. V. Perdue states:

"quando i carbonati di calcio nei terreni sono meno di ½ per cent, la regione e' pellagrosa; quando i carbonati di calcio sono fra ½ per cent e 1 per cent la regione e' raramente pellagrosa; quando i carbonati di calce sono 1 per cent o di piu' non esiste la pellagra."

"Where the carbonate of calcium in the soil is less than 1 per cent, the region is pellagrous; where the carbonate of calcium is between ½ per cent and 1 per cent, the region is slightly pellagrous; where the carbonate of calcium is 1 per cent or more, pellagra does not exist."

He has further shown that the surface water of the streams in the pellagrous area contains about 25 parts per million of silica and from 5 to 13 parts per million of calcium; that in the slightly pellagrous areas such as parts of Kentucky and Tennessee the silica and calcium in surface waters are very nearly equal, sometimes one and sometimes the other being in excess; that in the immune regions the silica averages about the same while the calcium is from 40 to 82 parts per million.

### THE TOXICOLOGY OF SILICA.

The toxicology of silica has been investigated by Hahnemann, Froissac, Goullon, Gross, Hering, Stapf, Wahle, Knorre, Ruoff, Baker and Robinson. Silica is toxic only in the colloidal state, that is, when it is finely divided and combined with ionized water. When in this condition it is readily absorbed and produces certain well defined and well known symptoms. The above named investigators determined the toxicology of silica by the administration of colloidal silica to human subjects. The work was commenced early in the 19th century, and while the greater part of it was done in the first half of that century, it has been continued and reviewed to date. The late researches confirm the work and results of the early investigators. The published reports of this work are too voluminous for reference in an article like this. They can be found in any well appointed medical library. The Index Catalog of the Library of the Surgeon General's office contains columns of references to them. The works containing the reports of this research in German, French, Italian and English fill several shelves in the writer's library. The literature is open to every conscientious investigator.

All the reports on the toxicology of colloidal silica are a complete clinical picture of pellagra. This is so striking, so complete, so convincing, that it requires a stretch of the imagination and a violation of credulity to believe that it has been overlooked in the later research.

This research has been repeated on the lower animals. It has long been well known that pellagra is a common disease of domestic animals in pellagrous regions. Pellagra in cattle was described at length by Giovanni Marchese in his Dizionario di Agricoltura, Vol. V, p. 18, in 1898. In 1914, Alessadrini and Scala quoted Marchese with approval and add that pellagra is common in the dogs and cats in Italy. They produced true pellagra in guinea pigs, rabbits, dogs and monkeys by subcutaneous and intraperitoneal injections of colloidal silica, and by giving it in drink and food. The post mortem examination of the animals dead of the disease confirmed the clinical picture.

In 1912, Scala showed that alcohol permeates organic tissue by the method of colloidal substitution; that each molecule of alcohol substitutes itself for a molecule of water; and that the process of hardening with alcohol is a process of colloidal deaquification. In 1916, Perdue stated: "The withdrawal of water has a marked effect on the nervous system. As in all drying processes, there is first excitement and hyperesthesia, followed as the drying progresses, by incoordination and paralysis. The same thing takes place in alcohol intoxication." Working at the same time, but independently, W. F. Lorenz, Special Expert, United States Public Health Service, after an exhaustive research and review of the mental symptoms in pellagrins states that "The similarity is all in the direction of the acute alcoholic psychoses." . . . "It is therefore suggested that in the instance of pellagra the symptoms do not result from an infection, but from a toxic substance of a chemical nature similar to alcohol in that it has specific deleterious properties."

Were an etiology to be suggested from the mental disturbances alone, the causes would fall among a group of agents similar to alcohol in that they are not products of bacterial or parasitic invasion of the body, but chemical intoxicants in the narrower sense."—Public Health Reports, Feb. 4, 1916, 221-246. Reprint No. 322. Public Health Reports.

All these findings are supported by the well known fact that the brains of persons dead of acute alcoholism absorb much more water than normal brains. That is, they absorb water to replace the water lost by the deaquification or substitution of the alcohol.—See Le Count and Nuzum, Jour. A. M. A. Dec. 16, 1916, page 1822.

### COUNTER RESEARCHES.

For the present, at least, it seems that the maize theory has been abandoned in this country. The same is equally true of the parasitic theory. Some recent researches have been undertaken to prove that pellagra is not transmissible. The rationale of such efforts is difficult to comprehend. How either a mineral intoxication or an autoxication could be transmissible is really beyond the comprehension of a truly scientific mind. The principal attack has been directed upon the findings and proof of Alessandrini and Scala.

For a number of years the Thompson-McFadden Pellagra Commission has centered its efforts at Spartansburg, South Carolina. This is a pellagrous region in the clay soils of the Piedmont Plateau. This is the very region surveyed by the United States Bureau of Soils and reported in the Soil Survey of the Campobella Area. In spite of all this, after describing the springs and wells of this region, Siler and Garrison, in the American Journal of the Medical Sciences, Aug. 1913, make the following statement:

"We realize that the water supply is not considered to be of any importance as a factor in the etiology of pellagra. In this study, however, we have endeavored to investigate, as far as was possible, all probable factors concerned in the epidemiology of the disease, and for this reason have considered the water supply. We have found nothing of any apparent significance in this connection."

The research of Alessandrini and Scala was done in Italv prior to August, 1914. Up to this time official and "authorized" medicine in America took its "kultur" from Germany. Therefore the findings made in the University of Rome were "unscientific." The United States Public Health Service undertook an official counter research to discredit the findings of Alessandrini and Scala. To conduct this experiment it selected a surgeon with a very German name. This so called experiment was conducted in the State of Mississippi. Twelve long term convicts from the State Penitentiary were the victims. It was predeterminated that pellagra was a "deficiency" disease. Scurvy was selected as the disease most readily confused with pellagra. The convicts were fed the same meal three times a day for nine months. This was a regimen calculated to kill the most hardy. Of course some of them took sick. Their sickness was pronounced pellagra. From this has followed the monstrous and gratuitous insult that the people of the South are underfed! This propaganda enjoys the prestige and frank of the Federal Government. It has deceived many and has greatly retarded the work of eradicating pellagra. Its wide acceptance in the South is unworthy the traditions of a proud people.

Another counter research was undertaken by Jobling and Peterson at Nashville, Tennessee. It was published in the Journal of Infectious Diseases, May, 1916, under the title "Epidemiology of Pellagra in Nashville, Tennessee." In this paper the following statement is made:

"We are unable to find that water has any relation to the disease. City water, and water from wells, springs and cisterns was used indiscriminately, without an apparent influence on the incidence of the disease. The well and spring water is "hard water," because of the presence of lime salts; this would tend to disprove the tendency advanced by Alessandrini and Scala that the disease is due to the presence of colloidal silicates, which are removed by adding lime."

(We purposely correct the spelling of the original.)

The general character of the above statement ignores the local and individual conditions in Tennessee, and takes no account of the fact that the river waters in Tennessee are evenly balanced in silica and calcium, and that there are many instances and localities in which the silica predominates.

All of these researches evidence the bias which is inseparable from a research undertaken to attain predetermined results. All show that poverty of scientific acumen which is such a disappointing characteristic of all the American research which has so long been a slave to the German method.

THE BIOCHEMISTRY OF PELLAGRA.

The colloidal state of matter and the colloidal reactions are of the utmost importance in the study of biochemistry. Nature looks with such favor upon the colloidal state of matter that she has chosen it exclusively as the vehicle for the presentation of all living things. All living tissue may be regarded as protoplasm combined with ionized water. The greater the amount of ionized water, the greater the dispersion of the proteid substance and the more nearly liquid the tissue. There is more water in the tissues in the embryonic state, and less water in each succeeding period of life. The process and mechanism of senility and of growing old consists in a deaquification or extrusion of ionized water. Time is of the essence of this process. The trajectory of life is indigenous and specific for every living organism at its inception. Senility and growing old are accomplished by the aggregation of colloids. As colloids aggregate they extrude water. This process in colloidal chemistry is called ageing of the colloid. The colloidal reactions of digestion are accomplished by the colloidal enzyme first attaching itself to the substance to be digested and then substituting water for the enzyme, thus setting the enzyme free to act to infinity unless changed, oxidized or swept out of the digestive tract. Toxic colloidal reactions are accomplished by substitution. Colloidal hydroxids substitute themselves for water in the proteid complex. This deaquifies the tissue. In turn colloidal acid hydroxids fix alkaline bases to the proteid substance and free the acids of the salts. These acids free in the tissues cause a true mineral acid intoxication. In this process the mineral colloids act like enzymes. They serve as the intermediate link, substituting themselves for water, fixing the alkalies, freeing the acid, and in turn being set free themselves to act to infinity unless neutralized by electrolytes or eliminated from the system.

The colloidal process of digestion, assimilation, metabolism and elimination are not the impossible complexes suggested by the involved and intricate formulae perpetrated by the chemists. The processes of nature are exceedingly simple. They are so simple that they are difficult for certain minds to understand. Their very simplicity presents an impossible problem to the mind trained in the modern school of research. The simple processes of oxidation and hydrolysis in the beta position do not suggest antigen, amboceptor, vitamines and the like. A study of these simples will tend to a rejuvenation of scientific thought. Simple, scientific, golden nuggets of truth lie in our pathway while the butterflies of theory and hypothesis float in the atmosphere of speculation. We are continually stumbling over the nuggets while we chase the butterflies.

If the proteid substance of the tissues be expressed by a conventional symbol as Pm, then colloidal proteid as found in all living matter may be expressed by the following formulae:

Pm(OH H)n and Pm(H OH)n, the bond with the hydroxyl or the hydrogen of the ionized water depending upon the electronic sign of the proteid. The strength of this bond will depend upon the strength of the positive or negative character of the proteid and will be measured by the amount of force necessary to accomplish dissociation.

The process of senility is a process of deaquification accomplished by the aggregation of colloids and the extrusion of ionized water. If there is proper elimination of wastes it is not attended by intoxication. Water is simply withdrawn and the tissue assumes a less dispersed phase.

The processes of intoxication are processes of deaquification accomplished by the colloidal substitution of other substances for the ionized water of the tissues.

In the formulae Pm(OH H)n and Pm(H OH)n, the process of senility only serves to decrease the value of n. Nothing is substituted in its place. On the other hand, the various processes of intoxication decrease the value of n by the valence of the substance substituted, and leave such substance in colloidal combination with the proteid substance.

In the normal human economy the process of senility is the ageing of organic colloids. The reaction of living fluids and tissues is alkaline. As ionized water is extruded in the process of growing old, there is a tendency to increased alkalinity so that in the aged the isotonic balance measured by a solution of sodium chlorid is one per cent or more, instead of 0.85 per cent of the normal adult.

In 1912, Scala, in a volume "In onore del prof. Angelo Celli nel 25 anno di insegnamento," pages 153 to 193 distinctly sets out the processes of aggregation of colloids and distinguishes the processes of deaquification by substitution.

In Revue Scientifique, May 30, 1914, Marinesco of Bucherest, in an article entitled "Mecanisme chimico-colloidal de la senilite et le probleme de la mort naturelle" discusses fully the difference between the processes of senility and autointoxication. At page 675 he says:

"Ces phenomenes de deshydratation des colloides comme expression de la vieillesse se retrouvent pendant l' evolution de tous les organismes vivants."

"These phenomena of dehydration of the colloids as an expression of growing old are found during the evolution of all living organisms,"

In 1903, Viola of Padua, in a study of the blood resistance of the aged and of certain autointoxications, showed that the two conditions were distinctly different.

The two colloids coming from clay and of interest in the etiology of pellagra are hydrated silica or silicic acid (SiO2) and hydrated alumina ($Al_2O_3$). In all ordinary clays, as in the rocks from which the clays are derived, the silica greatly predominates. In colloidal soils or conditions of great dispersion, as clay is always found in drinking water, these two colloids are incompatible, electrolytic and mutually precipitate each other. Since silica is greatly in excess, the alumina is nearly all precipitated and only the excess of silica remains to be the toxic factor in the etiology of pellagra. The average amount of silica in such drinking waters approximates 20 to 25 parts per million.

Colloidal silica is readily absorbed by both the animal and vegetable organism. If absorbed or administered hypodermically in considerable amount to the laboratory animals it kills in from 30 to 60 days. Ingested in drinking water its toxic course is generally chronic, being modified by the amount of silica in the water, the antidotes in the water and food, the amount of water taken, the susceptibility, resistance and elimination of the patient. Taken in great amount by plants, it stunts growth, dehydrates and hastens ripening.

Colloidal silica acts like an enzyme. The toxic effects are secondary and not direct. The silica acts as a colloidal intermediary, fixing the bases of the mineral salts in the protoplasm of the tissue cells, substituting water and setting free the acid of the salt. The silica is again set free and theoretically would act to infinity unless eliminated or rendered innocuous. Thus by a slow colloidal reaction, silica fixes the sodium, potassium, magnesium and calcium of the chlorids and sets hydrochloric acid free. It fixes the same minerals of the phosfates and sulfates and sets free phosforic and sulfuric acid. With each reaction a portion of water is also removed from the tissues. As the chlorids make up such a large part of the solids or salts, hydrochloric acid intoxication is one of the salient features of pellagra. Water exhaustion is the other important manifestation. It is suggested that silica also fixes the salts of the carbonates and that carbon dioxid is very mild and readily eliminated and plays no part in the intoxication. In fact, for this reason the carbonates are the most important antidotes to silica intoxication.

The foregoing observations have been proven by incontrovertible chemical formulae setting out the reactions with great exactness. These formulae are a basic contribution to the science of organic chemistry.

Two other proofs are added to the support of the correctness of the findings that pellagra is an acid intoxication.

To every true student of clinical medicine and of true materia medica, pellagra is an acid intoxication. More than this, the symptoms of a chronic hydrochloric acid intoxication or poisoning greatly predominate. The erythema, the desquamation following the erythema, the pyrosis, the thickening and induration of the pyloris, the atrophy of the intestinal musculature, the constipation and the final intractable diarrhoea, the physical weakness, the mental apathy, the vertigo, incoordination and paralysis, the final delirium tremens are all typical of hydrochloric acid poisoning and its attendant deaquification.

The second clinical proof is the fact that pellagra is readily cured by an antidote without change of diet, domicile, labor or sanitary environment. One of the most direct and efficient antidotes is a ten per cent solution of sodium citrate. One cubic centimeter is given hypodermically each day for fifteen to thirty days, then on alternate days. The citrate is oxidized in the tissues and antidotes the acid poison. Milk of magnesia, lime water, calcium lactate and other alkalies and antacids give similar results when given by the mouth, but may require a longer time. Recovery will be complete without other treatment, although many physicians also prefer symptomatic treatment of the symptoms.

Alessandrini and Scala in describing their treatment with sodium citrate repeat that they did not change the diet of the patient. In going over their work I find such expressions as the following:

"left in her own environment, at her own work, and with her scant and uniform diet"; "in her own environment and to her own alimentation"; while Dr. Ruggero Guerrieri, in reporting upon the treatment of a number of patients writes Alessandrini as follows:

"I am able to assure you that these same pellagrins have always lived during and after treatment, in the conditions of environment to which they were accustomed, poorly nourished as in the past."

Dr. Arturo Secchieri states that he kept his patients in identical conditions of environment, diet, clothing, work and domicile, and that they recovered under treatment with sodium citrate and without other medication.

In the Journal of the A. M. A., September 21, 1918, at page 954, Dr. Victor C. Vaughan, in discussing the etiology of pellagra states that he has made a careful study of all the publications on pellagra, including those of Dr. Goldberger and "those of his opponents." In this connection we submit that we have not adopted the role of "opponents" of Dr. Goldberger. Alessandrini and Scala were the first to establish upon a purely scientific basis the etiology of pellagra. They crowned their research by a successful method of treatment. The writer has been their collaborator in America. The work of Dr. Goldberger has been solely a "counter-research." He is the "opponent," if such a term must be used. We feel that humanitarian and altruistic medicine as well as pure science can afford such "opponents."

